# Perceptions of clinicians on promoting oral health care in an alcohol and other drug use health care service: A qualitative study

**DOI:** 10.1111/dar.14016

**Published:** 2025-02-18

**Authors:** Agnivo Sengupta, Kaniz Fatema, Tiffany Patterson‐Norrie, Shwetha Kezhekkekara, Prakash Poudel, Gilbert Whitton, Ravi Srinivas, Stephanie Hocking, Ajesh George

**Affiliations:** ^1^ Australian Centre for Integration of Oral Health, School of Nursing & Midwifery Western Sydney University Sydney Australia; ^2^ Ingham Institute for Applied Medical Research Sydney Australia; ^3^ Drug Health Services South Western Sydney Local Health District Sydney Australia; ^4^ Office of Research and Education Canberra Health Services, ACT Health Canberra Australia; ^5^ Oral Health Services South Western Sydney Local Health District Sydney Australia; ^6^ School of Dentistry, Faculty of Medicine and Health The University of Sydney Sydney Australia; ^7^ Centre for Alcohol and Other Drugs NSW Health Sydney Australia; ^8^ School of Nursing, Faculty of Science, Medicine & Health University of Wollongong Wollongong Australia

**Keywords:** alcohol drinking, AOD services, dental care, illicit drugs, oral health

## Abstract

**Introduction:**

Alcohol and illicit drug use is linked to a higher prevalence of oral health complications. However, substance use can lead to avoidance of dental services due to anxiety and competing health priorities. This study explores current knowledge, attitudes and practices of clinicians of an alcohol and other drug service regarding promoting oral health among their clients.

**Methods:**

Exploratory qualitative design using semi‐structured interviews with medical and nursing staff working as alcohol and other drug professionals in a Drug Health Service in South Western Sydney, Australia.

**Results:**

Three main themes were identified: perceptions of providing oral health care to clients; barriers to promoting oral health care; and recommendations for oral health integration in alcohol and other drug settings. Oral health concerns were identified as a significant issue for their clients within the alcohol and other drug setting. Considering a higher prevalence of oral health issues among clients, staff acknowledged that oral health interventions within alcohol and other drug settings would be beneficial. Barriers included access challenges for public dental services, affordability of private dental care, limited oral health training and time constraints. Staff recommends that training/resources are required to support staff in the provision of oral health promotion to clients.

**Discussion and Conclusions:**

Clients accessing alcohol and other drug services often have unmet oral health needs. The clinicians who participated in this study, are motivated to provide oral health promotion within this setting, however, they require training, resources (including allocation of time) and appropriate referral pathways to support provision of this service.

**Key points**

- Oral health concerns were identified as a significant issue for clients within the alcohol and other drug setting.
- Limited oral health training, time constraints, perceived challenges accessing public dental services, and affordability of private dental care for clients were barriers identified by clinicians to providing oral health promotion.
- These findings contribute valuable insight into the perceptions of clinicians around promoting oral health within alcohol and other drug services.

## 1 INTRODUCTION

Harmful or hazardous use of psychoactive substances, including alcohol and other drugs (AOD) contributes to several diseases, loss of productive years and premature mortality [1]. The World Drug Report 2023 stated that around 296 million people aged between 15 and 64 reported drug use in 2021, with 39.5 million experiencing drug use disorders [2]. In 2019, the National Drug Strategy Household Survey reported that approximately 43% of Australians aged 14 and over had illicitly used a drug at some point in their lifetime, and an estimated 16.4% had used an illicit drug in the previous 12 months [3].

Alcohol occupies a significant place in Australian culture [4]. The Australian Bureau of Statistics reported that one in four (5 million) people aged 18 years and over exceeded the Australian Adult Alcohol Guideline in 2020–2021 [4]. AOD use is associated with a range of psychiatric disorders, disability, and comorbidities [5]. Among various health problems associated with drug and alcohol addiction, oral health problems are highly prevalent, and thus require more attention not only by dentists but also by AOD care providers [6].

The use of AOD has a significant negative impact on oral health, resulting in a decline in oral health‐related quality of life [7, 8]. People using AOD often experience compromised immune systems and a preference for sugary foods and beverages, which further worsen their oral health problems [9]. This is manifested through dental diseases, including tooth decay, dry mouth and periodontal (gum) disease [7, 10]. Additionally, methadone (which is used for withdrawal management) along with other opioids and amphetamines, can reduce saliva flow, and enhance sugar craving and bruxism (jaw grinding) resulting in increased risk for tooth wear and decay [11]. Alcohol and tobacco use is also linked with increased dental problems while lower socioeconomic status and homelessness, which are more common among individuals who use AOD, can further impact oral health [12]. Epidemiological studies examining oral health in people who experience AOD use disorders have reported that higher prevalence of dental and oral mucosal diseases as compared to individuals who do not use AOD [13, 14]. A meta‐analysis of 28 studies conducted in 2017 reported that dental diseases were significantly higher in illicit drug users as compared to those who do not experience a substance use disorder [15]. The authors also reported that individuals who use AOD had an average of 3.5 more decayed teeth compared to those who did not but had lesser restorations [15]. Inadequate oral hygiene practices, including infrequent toothbrushing and flossing, and infrequent dental visits contribute to a higher risk of poor oral health among individuals experiencing a substance use disorder [13, 14, 16, 17]. Moreover, evidence suggests that individuals with substance use disorders undergoing withdrawal management experience both individual and structural barriers to access public oral health services, such as anxiety and fear of dentists, daily struggles to attend appointments and perceived stigma from dentists [17, 18]. Dentists also face several challenges when treating individuals experiencing a substance use disorder and often perceive, the management of this client group as demanding due to various factors like dental fear, difficulties in coping with appointments, and poor compliance to preventive measures [19, 20]. Dentists may sometimes overprescribe opioid medications, which are addictive and prone to abuse, which may ultimately lead to poor oral health outcomes [21, 22].

In Australia, AOD treatment services have two care systems: (i) the general health service system, where similar treatments are provided through general practitioners (GP), psychologists, general hospitals and welfare services; and (ii) the specialist system which offers various services such as withdrawal management, maintenance treatment and psycho‐social therapies [23]. After accessing an AOD treatment service, clients progress through withdrawal, rehabilitation, psycho‐social therapy and maintenance pharmacotherapy, as determined by their individual needs [23]. Considering the evidence from other studies that non‐dental professionals can actively contribute to promoting oral health among at‐risk populations, AOD clinicians also have an opportunity to initiate brief interventions [24, 25, 26]. These could include educating patients about oral health risks, conducting a brief oral health screening and initiating dental referrals. Results from a few studies suggest that individuals who use AOD rarely receive oral health information and education from AOD clinicians [14]. Currently, there is a dearth of evidence globally regarding the perceptions and practices of AOD clinicians towards oral health care, particularly within the Australian context. Therefore, we aimed to explore the knowledge, attitudes and practices of AOD clinicians regarding promoting oral health among AOD clients.

## 2 METHODS

### 2.1 Design, setting and population

We used an exploratory qualitative design involving semi‐structured interviews with AOD clinicians working across a large Drug Health Service in Greater Sydney, New South Wales, Australia. The AOD clinicians work across a range of services, including opioid treatment, outpatients, substance use in pregnancy and parenting, general practice advice and support, hospital consultation and liaison, withdrawal management and blood‐borne virus treatment. This area of Sydney was particularly chosen as the population living here is culturally and linguistically diverse, and a significant proportion experience a high level of socioeconomic disadvantage and homelessness [27]. A health report prepared by the South Western Primary Health Network reported a high prevalence of illicit drug usage in some parts of South Western Sydney, where the population is estimated to increase by 29% (from 110,193 people to 141,673 people) by the year 2031 [28].

Purposive sampling was used to recruit participants. Clinicians and other health professionals working in the Opioid Treatment Programs, the Harm Reduction programs, Counselling, Specialist Medical Consultation, Withdrawal Management Program, and Assertive Youth Services were invited to participate in the study. Study flyers were electronically distributed to all staff at each of the facilities by research coordinators working within the service. Interested participants were directed (via flyers) to contact the recruitment champion (PP and KF) who checked for eligibility for recruitment. Participants were then handed over to the appropriate study investigator (TPN) where rapport was established and a time was organised for the interview to take place.

### 2.2 Data collection

Interviews were individually conducted by one researcher [TPN (MND)] with AOD clinicians (doctors and nurses) over a videoconferencing platform (Zoom) using an interview guide (see Supporting Information) that was informed by a previous review in this area and experts in the field [19]. Interviews were 15–20 minlong and were audio‐recorded. Written informed consent was obtained from all participants prior to the start of the interview. Audio files were transcribed using a professional transcription service.

### 2.3 Data analysis

All transcripts were then uploaded to a qualitative data management/analysis software (NVivo 12 pro) [29]. A hybrid (deductive and inductive) approach to thematic analysis was conducted to identify and analyse contextual patterns and themes within the data [30]. Initially, the transcripts were carefully reviewed multiple times to gain familiarity with the data and to record initial ideas. Using a deductive approach a priori coding framework was developed, informed by the semi‐structured interview guide to identify the major themes. Data was then coded into the major themes. Two researchers [SK (MPH) and AS (MPH)], who were trained in qualitative research, then independently recoded and regrouped the data using an inductive approach informed by Braun and Clarke to identify sub‐themes [31]. The coding structure was then further refined by two other researchers [AG (PhD) and KF (PhD)]. Team meetings were then organised to discuss similarities and differences in the themes and interpretations and a consensus was achieved. An inductive approach guided the consensus meeting to help the team understand the perceptions and barriers of AOD clinicians towards promoting oral health. The findings were presented with the use of pseudonyms for doctors (D) and nurses (N).

### 2.4 Ethical considerations

This study received ethics approval from South Western Sydney Local Health District Research and Ethics Committee (2021/ETH12072). The audio recordings and transcripts were stored on a password‐protected computer as per institutional and ethics committee requirements. Participants were deidentified throughout the transcriptions to ensure anonymity and confidentiality, and numeric pseudonyms were used in the quotes from participants.

### 2.5 Trustworthiness

Various methodological techniques were employed to enhance the trustworthiness of the study. Interviews were conducted by a researcher (TPN) trained in qualitative research methodologies. Debriefings were organised with another researcher (AG) to discuss completeness of the data and identify any potential new areas to explore in subsequent interviews and continued until analytical sufficiency was achieved [32, 33]. A professional transcription service was used to enhance accuracy of the verbatim transcriptions of the audio recordings. Two members of the study team (SK and AS) independently checked the data for accuracy and performed the coding. Coding consensus was achieved with the whole team. Adequate information about the participants, study settings, and data collection are provided in the results and the findings are supported by direct quotes from the participants.

## 3 RESULTS

Sixteen participants were interviewed, of which seven were doctors and nine were nurses. No participants dropped out prior to interview. All participants worked in drug health services in various positions including addiction specialists and trainees (*n* = 3), medical officers (*n* = 3), psychiatrists (*n* = 1), registered nurses (*n* = 1), clinical nurse consultants (*n* = 7) and nursing managers (*n* = 1). Two of the doctors also had general practice experience. Participants had a mean (SD) age of 47.29 years (±12.75) with varied clinical experience in drug health services (range 1–35 years). Most of the participants were female (*n* = 9). The thematic analysis of the data resulted in three major themes and eight sub‐themes, which have been outlined in Table 1.

**TABLE 1:** Themes and sub‐themes.

Themes	Sub‐themes
Perception of providing oral health care among clients	High prevalence of poor oral health and its impact
	Clinical practices regarding oral health
Barriers to promoting oral health care in AOD settings	Limited oral health training and time constraints among clinicians
	Perceived lack of priority, accessibility and affordability of dental services for clients
Recommendations for oral health integration into AOD settings	Oral health education and screening
	Tailored resources and referral pathways

Abbreviation: AOD, alcohol and other drugs.

### 3.1 Perceptions of providing oral health care among clients

#### 3.1.1 High prevalence of poor oral health and its impact: ‘nine out of ten patients have oral health issues’

Most participants reported that majority of their clients that presented in their services had oral health issues and needed dental care. This was mainly attributed to the use of alcohol and drugs, low nutrition and a general lack of awareness about oral hygiene.

‘I think most of our clients need dental work … So, I think most of our clients who do have an extensive drug history, have dental issues more often than just.’ (N8)

‘I'd say in the process that I just described to say nine out of ten patients have oral health issues.’ (D2)

‘I remember this one comment, that one of the patients said that, look, I would be using this toothbrush after seven or eight years.’ (D3)

Most of the doctors and nurses recognised the role of oral health for their clients, citing that it ‘has a big impact on their self‐esteem’, and that having poor oral health was also a reason for experiencing stigma from the community. Some people may have a belief that a facial appearance reflecting poor oral health (like missing teeth) is directly caused by AOD use and/or AOD service treatments, such as methadone maintenance treatment; and this brings into play all the negative feelings that come from being labelled ‘an addict’.

‘It [oral health] really has a big role and big impact on their self‐esteem, on the belief of being accepted in the community because even though they are on the program [methadone] and they are kind of having their life back on track, having very poor oral health, it's always being picked and kind of stigmatising them anywhere they go.’ (D7)

In this context, one clinician also recognised the importance of oral health in securing and retaining employment in this population. It was also mentioned that poor oral health is a risk factor for serious health complications as it might lead to sepsis or other chronic diseases.

‘So, there are just a few prominent things, not to mention the psychosocial aspects, the stigma, the shame, the difficulty getting a job if you've got a mouth like that.’ (D4)

‘If you don't look after your teeth, you're more likely to die from a heart attack much, much earlier. If you don't look at the teeth, you [are] very open to symptoms like becoming septic. If you don't look after your teeth, every part of your body can become infected, including your brain.’ (N4)

Two doctors and two nurses mentioned that opioid medications such as methadone might worsen oral health‐related quality of life.

‘But I do know that people that have that are on methadone can have more problems with their teeth. I think it might be related to saliva or something like that.’ (N9)

However, one clinician reported that although some clients develop misconceptions that methadone is the only reason for their poor oral health, they should also be counselled on the debilitating effects of substance use.

‘When they come in to [see] me and they've been on methadone for six months and say “look at my teeth”, you know, “look what methadone has done to my teeth”. And I say, “probably this is the first time you've looked in the mirror for the last 18 years. Now that you're not running around seeking drugs and relatively stable, you've actually noticed what's been going on for two decades there. And if you want to blame methadone or well, but don't forget to blame heroin, which was working on that for a long time.” So, you see those things all are addressable in counselling where you stratify what the risks are.’ (D1)

#### 3.1.2 Clinical practices regarding oral health: ‘I don't always do it’

Most of the doctors and nurses reported that they do not initiate discussions about oral health with their clients, in general, unless being prompted by the client about an oral health‐related issue.

‘It's truthful to say I don't always do it [talk about oral health]. And it's really good doing this interview because I'm aware of the times that I don't always do it, even though, you know, if you ask me, I go, “yeah, it's really important”.’ (D5)

Most nurses mentioned that when oral health complaints come up, they are mostly involved in referring cases to dentists or GPs.

‘They'll always complain about, you know, they've got toothaches or infections or anything like that. And then we would recommend to get a referral to the dentist … And if they wanted some assistance with that, we can sometimes give numbers to any services that we do know that assists patients with accessing oral health care or we would suggest to go to GP.’ (N7)

A few doctors noted that they typically conduct a brief oral health examination during their routine assessments, which not only ensures client satisfaction but also addresses any oral health concerns.

‘So, I'll do the routine review, which will entail talking about the substance use, talking about usually the social aspects around it, how it affects their health, and also the physical and mental health aspects. And then usually towards the end we'll go through an examination, examine them neurologically, and then ask if we can have a look in their mouth, just as a simple oral health screening. And it makes them happy that for us to have a look and are eager to show that they've got these issues …’(D2)

A couple of nurses reported they were proactive regarding oral health and made an effort to ask about oral health at their first meeting with a client.

‘We know for a fact that oral health and the rest of your body suffers if we don't look after it … Every patients that sees me, I look at their teeth.’ (N4)

‘I try to have that really non‐judgmental approach in terms of, you know, often phrased it in. You know, tell me a little bit about your teeth.’ (N3)

A few doctors mentioned that they provide oral health education to their clients in the form of promoting toothbrushing habits by educating them about brushing techniques.

‘Make sure you've got that toothbrush up [and] you're spending 2 minutes really massaging them, then it should feel like this … instead of scratching. That seems to get across to people what the sensation of brushing gums should be like.’ (D4)

### 3.2 Barriers to promoting oral health care in AOD settings

#### 3.2.1 *Limited oral health training and time constraints among clinicians:* ‘we've never been trained …’

Most clinicians cited time constraints to be the main reason for being unable to broach the topic of oral health with their clients. Two doctors mentioned the importance of taking a holistic approach with drug health clients and that there would always be several issues to cover during a consultation.

‘It [asking about oral health] can fall off my own radar because there are so many issues that demand attention … And I do work very holistically, and a lot of things out there have to be popped into that holistic space … I think there's a load of issues that need to be covered and that relates to time.’ (D5)

Of all participants, three clinicians reported that their knowledge about oral health was limited as they never experienced any dental training during their medical courses. One of them also went on to say that they felt ‘under‐resourced’ and ‘under‐informed’ in relation to the guidelines around dental management.

‘I'd say as a medical professional, I think we're limited in the extent we don't know the complexities of beyond the simple dental emergency. So, I would say, the times we are probably over treating things in terms of antibiotics and over treating things in terms of pain management, just because it is a bit of black box understanding, what's the underlying root of the problem. So, in that way, I do feel as medical professionals in general, we're under‐resourced or under‐informed as to the guidelines around dental management.’ (D2)

‘Well, I think the big issue is that and I blame medical faculties—we've never been trained in oral health and we've never had lectures on oral health. There are so many areas that we don't get trained in. We get made to do incredible learning with stuff that we'll never see or never use in our lives. But when it comes to sort of having a dental day as part of your medical training hasn't occurred and it's relevant, it's certainly relevant.’ (D1)

#### 3.2.2 *Perceived lack of priority accessibility and affordability of dental care services for clients:* ‘I think that*'*s kind of low down on their priority list …’

Most clinicians mentioned that their clients would generally refrain from accessing dental treatments. They mentioned that this was mainly due to the lack of prioritisation of oral health, as they would mostly have other pressing issues to deal with, such as homelessness or drug dependence.

‘Maybe in their own mind, if they haven't got acute pain from a tooth problem. *If my housing is not good and I don't have a roof over my head and I'm worried about my kids … or I'm really not on top of this ice dependence yet*. I just think it [oral health] slips down their own internal priority list.’ (D5)

‘It's not that they are ignorant. No, it's just that it's not a priority for others. But keeping the kids is more of a priority.’ (N4)

Another frequently cited barrier was the high cost of dental care/treatments. It was also mentioned that this issue is more prevalent to clients coming from a low socioeconomic background, increasing their financial vulnerability.

‘I think that's kind of low down on their priority list and with them a lot of dental, you know, being private and hard to access. Obviously, the cost is very difficult for so many of our more vulnerable clients.’ (N8)

‘And especially now, the hurdles are getting harder because, as you know, most private dentists ask astronomical fees [which is] totally unattainable by our clientele.’ (D1)

‘Do you know how long it takes to get your teeth fixed? Far too long. Do you know how much it costs to get your teeth fixed? Far too much.’ (N4)

Even services like the public dental services that were free for low socioeconomic communities were difficult to access for clients. Some clinicians were even unsure of existing public dental referral pathways and how to access them.

‘There's been a couple of times that I tried to ring like it's a centralised number or something. Some public health … and it went basically no where.’ (N1)

‘I don't remember any referral process here [for dental]. Maybe something that we need to have as well.’ (N2)

‘We don't have a system to refer people specifically into public dental and oral health care.’ (D4)

### 3.3 Recommendations for oral health integration into AOD settings

#### 3.3.1 Oral health education and screening: ‘I think it is our responsibility to be screening and identifying conditions …’

Most physicians mentioned that it would be highly appropriate for them to provide oral health education in the AOD clinics, as they thought that looking at their clientele's health and wellbeing holistically would be an effective way of identifying health conditions and preventing further problems.

‘I think it is our responsibility to be screening and identifying conditions, of course, not necessarily knowing the best course of treatment, but at least knowing the care pathways and how to get people to care in the best way.’ (D1)

‘It's very appropriate because we are trying here to have this holistic approach to the patient's well‐being … we are trying to address the reason and we're trying to help them have a better lifestyle.’ (P7)

A couple of doctors mentioned that oral health education should be provided after the acute issue of drug dependence has passed and once the clients are stable.

‘Once the acute issue of the drug issue is beginning, you know that people are more physiologically stable to include it in maybe, package of information about general health issues and preventative health … So, I just want to talk to you about immunisation and so that dental health is part of a little package of really obvious preventative health things.’ (D5)

‘I explained why it's [oral health] important and can affect their health. But ultimately, I do defer to them to prioritise that because often they have competing issues and it's about seeing where that fits in relation to those other issues as to what they pursue, whether it be housing issues or domestic violence issues or substantive issues or other health issues. So, I can only raise it as an issue if they find it important and they'll usually ring.’ (D2)

Half of the nurses also mentioned that integrating oral health into routine assessment might be an effective way of asking about oral health issues.

‘I think an [oral health] assessment would be good … I mean, if it's just kind of asking, “do you have any oral health issues and can I help you link you into a service?”., I think that's completely appropriate.’ (N8)

‘… it would be good anyway to have that incorporated in the initial assessment so that information is passed on to the client. And then we do the follow up reviews.‘(N2)

A few doctors and nurses also suggested that oral health education and screening should be provided by all health professionals who see clients in AOD settings. This would ensure consensus in delivering oral health promotion to clients.

‘It should be everyone. This is a service where we have a multidisciplinary approach and we sit and we discuss patients. So, if I'm overlooked at all, I missed it. The nurse or the caseworker will come and raise it and we'll see how we can help it.’ (D7)

‘I think they should all be able to do it. Everyone can have a look and ask a question … Everyone's got a responsibility.’ (N4)

#### 3.3.2 Tailored resources and referral pathways: ‘We don't have a system to refer people’

Most physicians suggested that having resources such as brochures, patient education videos, and protocols or guidelines for dental referrals would be helpful in promoting oral health.

‘I think having a brochure or a poster might be useful, but again, it's difficult because we have quite a few things here. But I think some sort of poster at least for the dental clinic information. I think it would be quite handy to have in a waiting room and just have people know that the services that they can easily access. So, I think something simple just to make it a friendly environment where people know that they can approach it, it's a starting point.’ (P2)

A few nurses emphasised ‘it's very much about just having the conversation’ (N1). However, providing a resource that was ‘clear and concise and simple …’ (N1) and ‘given in a sensitive way…’ (N3) was also important.

Stating that current referral pathways for dental treatments are associated with long waiting periods in the public dental hospitals, most physicians and nurses stressed on the importance of having a seamless referral pathway between the AOD clinics and public dental clinics.

‘So, I think we could have … a sheet of how to get into the dental referral stuff, because we all know that the system has a lot of delay, you know, and particularly it doesn't really fast track our clients.’ (P1)

## 4 DISCUSSION

This study explored the knowledge, experiences, and perceptions of AOD clinicians (doctors and nurses) regarding oral health among their clientele. To our knowledge, this is the first study to explore the perceptions of doctors and nurses in an Australian AOD service setting The AOD clinicians in this study recognised that poor oral health was a significant problem among their clients and these findings reaffirm current knowledge in this area. Previous literature has shown that individuals who have a substance use disorder have more oral health problems, high unmet dental treatment needs, and reduced oral health‐related quality of life compared to other populations [20, 34]. Similar to other studies [14], the findings also show that oral health concerns are not often discussed by AOD clinicians during the course of hospital admission or at treatment services due to various barriers.

One of the key barriers identified was the perceived lack of accessibility and affordability of dental care services for clients. Private dental services were viewed as highly cost‐prohibitive for clients, which has been echoed in other studies where low prioritisation of dental care is attributed to lifestyle and socioeconomic factors [17, 35]. As private dental services are unaffordable for many people who have substance use disorders, more clients undergoing rehabilitation or medication will need AOD clinicians to provide basic oral health advice and appropriately refer clients to public dental services [36]. This indicates the importance of integration of oral health protocols in general assessments and creating referral pathways through which AOD clinicians can easily refer clients to public dental services.

Some AOD clinicians in our study, however, also acknowledged that they were not aware of existing referral pathways to public dental services. In New South Wales, Australia, the Oral Health Fee for Service Scheme provides free dental care to eligible clients with substance use disorders through public dental services [37]. Due to limited capacity and long waiting lists, clients are issued vouchers to receive dental care from private practitioners registered under the scheme. However, clients often face challenges such as finding suitable providers, understanding cultural needs, and miscommunication [38]. Additionally, there is a lack of data on the uptake of the Oral Health Fee for Service Scheme among this population, and potential issues include choosing providers, making appointments and lack of trauma‐informed care practices [38]. As public dental services in Australia prioritise emergency dental needs [39], it indicates the need for guidelines and simple, explicit protocols for advice, screening, and referrals [24]. Recently, a Parliament Inquiry into the provision of and access to dental services in Australia invited various submissions from national and state‐level peak bodies and organisations, which discussed the unaffordability and various other systemic barriers to access dental treatment experienced by various socioeconomically disadvantaged populations like AOD clients. Strategies such as integration of oral health care into general health care, providing extended coverage under Medicare (universal health insurance scheme for all Australians), and boosting resources for public dental services were some of the initiatives discussed [40]. These strategies need to be progressed further and implemented to address this important barrier to oral health care for AOD clients. It is equally important to ensure dental referral pathways provide appropriate care as AOD clients can experience overt or subtle stigmatisation in dental settings [41, 42], which may further deter them from seeking dental treatment.

Another important barrier was the lack of oral health knowledge and training among AOD clinicians. One of the contributing factors is the limited oral health training provided in undergraduate medical/nursing programmes, which was clearly highlighted in the study findings and previous research [43, 44, 45]. A survey of 132 general practitoner training programme directors in UK revealed that the majority of programmes (71.2%) did not provide any structured oral health training and very few (10%) trainees were undertaking clinical placements relevant to oral health [44]. Similarly, a global survey of the deans at medical, nursing, and pharmacy schools in universities across Canada, the United States, Europe, Asia, Australia, and New Zealand found that the majority (59.6%) rated their curricula in oral‐systemic health as inadequate [43]. These findings reinforce the need for greater foundation knowledge in oral health among undergraduate medical/nursing students to better prepare future AOD clinicans. Doctors in our study were quite receptive of education sessions or modules that contributed to continuing professional development points; therefore, developing continuing professional development training courses for AOD health professionals on oral health care could be an additional strategy to address this gap in knowledge and clinical practice [46]. In addition, our results suggest that AOD clinicians would benefit from oral health promotion tools to assist them to advocate for oral health care, undertake oral health screening, and provide referrals to appropriate dental services. It is also important that AOD clincians are aware of accessible and affordable dental services that can be shared with clients. Studies conducted in other healthcare settings in Australia have shown that training non‐dental professionals like midwives in oral health promotion has been effective in changing practice and improving patient outcomes through oral health education, screening and referrals [25, 26].

While some participants in our study advocated for oral health among their clients, other doctors and nurses identified the lack of time to be the main barrier in broaching oral health with their clients. Consistent with our findings, a lack of time has been frequently reported as one of the barriers to integrating oral health care into practice [47, 48, 49, 50]. Limited time during consultations emphasises the need for development of oral health education and screening resources that are concise, memorable, and conveyed at a low literacy level. This is also reflected in a recent scoping review which highlights the absence of existing interventions, models of care, and appropriate resources to promote oral health in the AOD setting [24]. Such tools (in the form of screening tools) should help clinicians in assessing the extent and nature of dental care needs of a client which further expedites the referral process.

Lastly, the need for interprofessional collaboration was highlighted in the study findings and has an important role in AOD settings wherein doctors, nurses, and case managers can work closely with dental practitioners to bridge the gaps in current clinical practices [11]. Moreover, our study results further suggest that there is a crucial need for oral health education among all healthcare professionals who see clients in an AOD clinic, which could include the ‘nurse or the caseworker’. Effective coordination or integration of oral health services and other health and social services can be highly effective in providing help and treatment services to this vulnerable population group [21, 45]. This could help in preventing people with substance use disorder from falling through the cracks. For example, the North Richmond Community Health Service, Australia, delivered low‐cost oral health care through public oral health practitioners providing assessments, preventative treatment and dental referrals at a medically supervised injecting room in the North Richmond Community Health Service [51], resulting in a high uptake of this programme. Additionally, in the USA, the FLOSS programme, which offered comprehensive oral health care (through university dental staff and students) to a sample of patients with substance use disorder and significant dental needs, reported improved treatment outcomes in relation to drug abstinence, completion of withdrawal rehabilitation treatment and employment [52]. These examples highlight that collaborations between both AOD and oral health clinicians is feasible and necessary in future models of care to improve the oral health of clients [54–57].

## 5 LIMITATIONS

Although we were successful in recruiting doctors and nurses from a service in a local health district that provides care to a significant number of AOD clients in the Greater Sydney Area, our study has a few limitations. First, we were only able to recruit a small number of participants from one service. In addition, we were unable to capture the views of clinicians from other AOD services across Australia. Therefore, our findings need to be interpreted with caution and larger studies are required in this area to confirm our findings. Nevertheless, our study has provided a valuable insight into this important yet underresearched area of care for AOD clients.

## 6 CONCLUSION

Our study has highlighted the limited emphasis being placed on oral health by doctors and nurses in an AOD service in Australia despite the high prevalence of poor health among clients. AOD clinicians can play a vital role in providing oral health education, screening, and referral if current barriers are addressed. Oral health training in undergraduate courses and through professional development programmes are needed to capacity‐build AOD clinicians in this area along with appropriate oral health resources that take into account their time constraints. Additionally, there is a need to have appropriate dental referral pathways that are affordable and accessible for AOD clients. These strategies could be further supported by interprofessional collaboration between AOD staff, social workers, and dental professionals to ensure comprehensive oral health care is provided to this priority population. This study has provided a valuable platform to develop tailored strategies that address the practice gaps among AOD clinicians and unmet oral health needs of clients.

## AUTHOR CONTRIBUTIONS

Each author certifies that their contribution to this work meets the standards of the International Committee of Medical Journal Editors. AG, PP, SH and RS conceptualised the study and the research design. KF and PP coordinated recruitment of participants. TF completed data collection. AS, SK, KF and AG analysed the data and all authors (AS, KF, TPN, SK, PP, GW, RS, SH, AG) contributed to the interpretation of the data. AS drafted the first version of the manuscript, which was further refined by AG and then reviewed by all authors for important intellectual content. The final version has been approved by all authors, and all authors have agreed to be accountable for all aspects of the work.

## FUNDING INFORMATION

This study was funded by a partnership grant from Drug Health Services, South Western Sydney Local Health District, and Western Sydney University.

## CONFLICT OF INTEREST STATEMENT

The authors declare no other conflict of interest.

## Supporting information

Supporting Information.

## Data Availability

The data that support the findings of this study are available from the corresponding author upon reasonable request.
