# Supplementary material for: Perceptions of clinicians on promoting oral health care in an alcohol and other drug use health care service: A qualitative study
Source: Drug Alcohol Rev. 2025 Feb 18;44(3):742–53. doi: 10.1111/dar.14016 (PMC11886495; doi:10.1111/dar.14016)
Supplement: Supplementary file 1 — Supporting Information. [file DAR-44-742-s001.docx]

**Interview guide – AOD clinicians**

1. What do you know about the topic of oral health in the context of Alcohol and Other Drugs (AOD) settings? Do you think oral health is important for AOD clients? (why/why not?)
2. Do you currently talk about oral health with your clients? (What do you exactly say, when do you say this. Is it part of your assessment (any screening form/process)? Do you initiate the topic or patients? Elaborate.
3. Do you recall…whether you have encountered patients with poor oral health/oral health problems in the AOD settings? (How often..? What type of oral health complaints do you usually encounter? How many patients out of 10 patients would have poor oral health?)
4. What do you do when you identify patients with oral health problems? (Do you initiate referral? How do you make this referral? If so do you follow up at the next appointment? If not, are there any established referral pathway to oral health services?)
5. Do you think it is appropriate to provide oral health education, assessment and referral in the AOD settings? Elaborate (why/why not- how this is useful). (Who would be best to provide oral health care services to patients- medical/nursing allied health clinicians? (Any reasons behind such views?)
6. What are your views about AOD clinicians (medical officers/specialists/nurses) providing oral health education, assessment and referrals to clients? Would there be any barriers (time constraint, limited education)
7. Would you think whether patients be comfortable to discuss oral health issues? Do you think they will be responsive to advice provided by AOD clinicians? What do you think could be some of the barriers for patients receiving oral health education, assessment and referrals? Is this important for them –other issues to get treatment- such as access, cost of dental care etc.)
8. In relation to educating patients. What do you think would be the best way to provide oral health education to patients? (Verbally or using brochure etc.) Do you currently have any oral health promotional resources? What sort of resources would be useful?
9. Do you feel that you have the adequate knowledge and confidence to provide oral health education, assessment and referrals to patients? (if not, elaborate what would be required?)
10. If an oral health education/training program were to be developed for medical officer/specialist/nurses what information do you think should be included in the program? If a program is developed how you do think would be the best way to deliver this program? Would it be useful if it was a CPD program?
11. Are there any other comments or suggestions you would like to make on this topic?
